# Paternal diet induces transgenerational epigenetic inheritance of DNA methylation signatures and phenotypes in sheep model

**DOI:** 10.1093/pnasnexus/pgac040

**Published:** 2022-04-14

**Authors:** Camila U Braz, Todd Taylor, Hadjer Namous, Jessica Townsend, Thomas Crenshaw, Hasan Khatib

**Affiliations:** Department of Animal and Dairy Sciences, University of Wisconsin-Madison, Madison, WI 53706, USA; Department of Animal and Dairy Sciences, University of Wisconsin-Madison, Madison, WI 53706, USA; Department of Animal and Dairy Sciences, University of Wisconsin-Madison, Madison, WI 53706, USA; Department of Animal and Dairy Sciences, University of Wisconsin-Madison, Madison, WI 53706, USA; Department of Animal and Dairy Sciences, University of Wisconsin-Madison, Madison, WI 53706, USA; Department of Animal and Dairy Sciences, University of Wisconsin-Madison, Madison, WI 53706, USA

**Keywords:** transgenerational epigenetic inheritance, paternal effect, DNA methylation, epigenetic reprogramming

## Abstract

Transgenerational epigenetic inheritance (TEI) requires transmission of environmentally induced epigenetic changes and associated phenotypes to subsequent generations without continued exposure to the environmental factor that originated the change. TEI is well-established in plants and *Caenorhabditis elegans*; however, occurrence in mammals is debated and poorly understood. Here, we examined whether paternal diet from weaning to puberty-induced changes in sperm DNA methylation that were transmitted to subsequent generations. Over 100 methylated cytosines, environmentally altered in the F0 generation, were inherited by the F1 and F2 generations. Furthermore, the F0 paternal diet was associated with growth and male fertility phenotypes in subsequent generations. Differentially methylated cytosines were correlated with gene expression. Our results demonstrate that some sperm methylation sites may escape DNA methylation erasure and are transmitted to subsequent generations despite the 2 waves of epigenetic programming: in primordial germ cells and in embryos after fertilization. These results advance our understanding of the complex relationships between nature and nurture.

Significance Statement TEI, well-documented in plants and nematodes, is minimally documented in mammals. To meet the requirements of TEI, environmentally altered epigenetic marks and associated phenotypes must be transmitted at least to the first nonexposed generation. This study investigates the effect of paternal diet on sperm DNA methylation across 3 generations and the phenotypic outcomes of diet exposure. For the first time, our results demonstrate the inheritance of over 100 epigenetic marks and the alteration of reproduction and growth traits by a grandpaternal diet.

## Introduction

The interplay between the environment and genes is an old debate that started with the spermism theory of Pythagoras in 530 BCE and continued in the 18th and 19th centuries through Lamarck's and Darwin's theories (1). More recently, this debate has been reinvigorated by progress in epigenetic research methodologies. Environmentally induced epigenetic changes, including DNA methylation, histone modification, and noncoding RNAs (2, 3), can be potentially transmitted to the next generation via the germline (4). The transmission of epigenetic marks from parents to offspring when all generations involved are directly exposed to the inducing agent is called intergenerational epigenetic inheritance (5). In contrast, transgenerational epigenetic inheritance (TEI) refers to the transmission of epigenetic changes across multiple generations without persistent exposure to the environmental factor that initiated the change (5). Despite substantial evidence of TEI in plants, *Caenorhabditis elegans*, and fruit flies, data to support this phenomenon in mammals remain limited and controversial due to multiple challenges associated with studies of epigenetic reprogramming and the lack of reproducibility across studies (6). In mammals, 2 distinct waves of epigenetic reprogramming erase DNA methylation and reset histone modifications: one in the development of primordial germ cells and the second in development of the embryo after fertilization (7). However, despite these 2 waves of epigenetic reprogramming, imprinted control regions and transposable elements resist reprogramming. The question of whether other genomic regions show TEI in mammals has not been fully answered.

Indeed, to meet the requirements of TEI, both environmentally induced epimutations in the germline and associated phenotypes must be identified across subsequent generations, at least through the first nonexposed generation (8, 9). Only partial evidence of TEI has been reported in mammals, as no studies have met both these criteria (5, 8–11). In some studies (12, 13), only 1 generation was reported for DNA methylation changes after an environmental intervention. Sadler-Riggleman et al. (14) exposed gestating rats to endocrine disruptors and studied the effects on DNA methylation, gene expression, and noncoding RNA in Sertoli cells of F1, F2, and F3 generations. Differentially methylated regions (DMRs) between treatment and control samples were identified in only the F3 generation. Other studies reported changes in phenotypes across generations that were not associated with inherited epigenetic changes. Ng et al. (15) found that paternal high-fat diet consumption was associated with increased insulin sensitivity, body weight, and adiposity in the F1 generation. Epigenetic changes were not reported in this study. A breakthrough study on nutritional effects in the viable yellow agouti mouse showed that supplementing pregnant female mice with methyl donors resulted in DNA methylation changes of the agouti locus associated with coat color and decreased obesity in the offspring (16). However, whether the effects reported are truly TEI or are due to different intrauterine exposures is not clear (7). Furthermore, no evidence of TEI was reported in the viable yellow agouti mouse model beyond the F2 generation (17).

These studies contributed significant advancements in environmental epigenetics research; however, most results represent intergenerational, not transgenerational effects. Furthermore, most of the research on dietary modifications affecting epigenetic inheritance has focused on the maternal diet during pregnancy. We hypothesized that exposure of males from weaning to puberty to the methyl donor, methionine, would alter sperm DNA methylation across multiple generations and impact the phenotypic performance of the offspring.

## Results

### Transgenerational inheritance of methylated cytosines

**Fig. 1.**
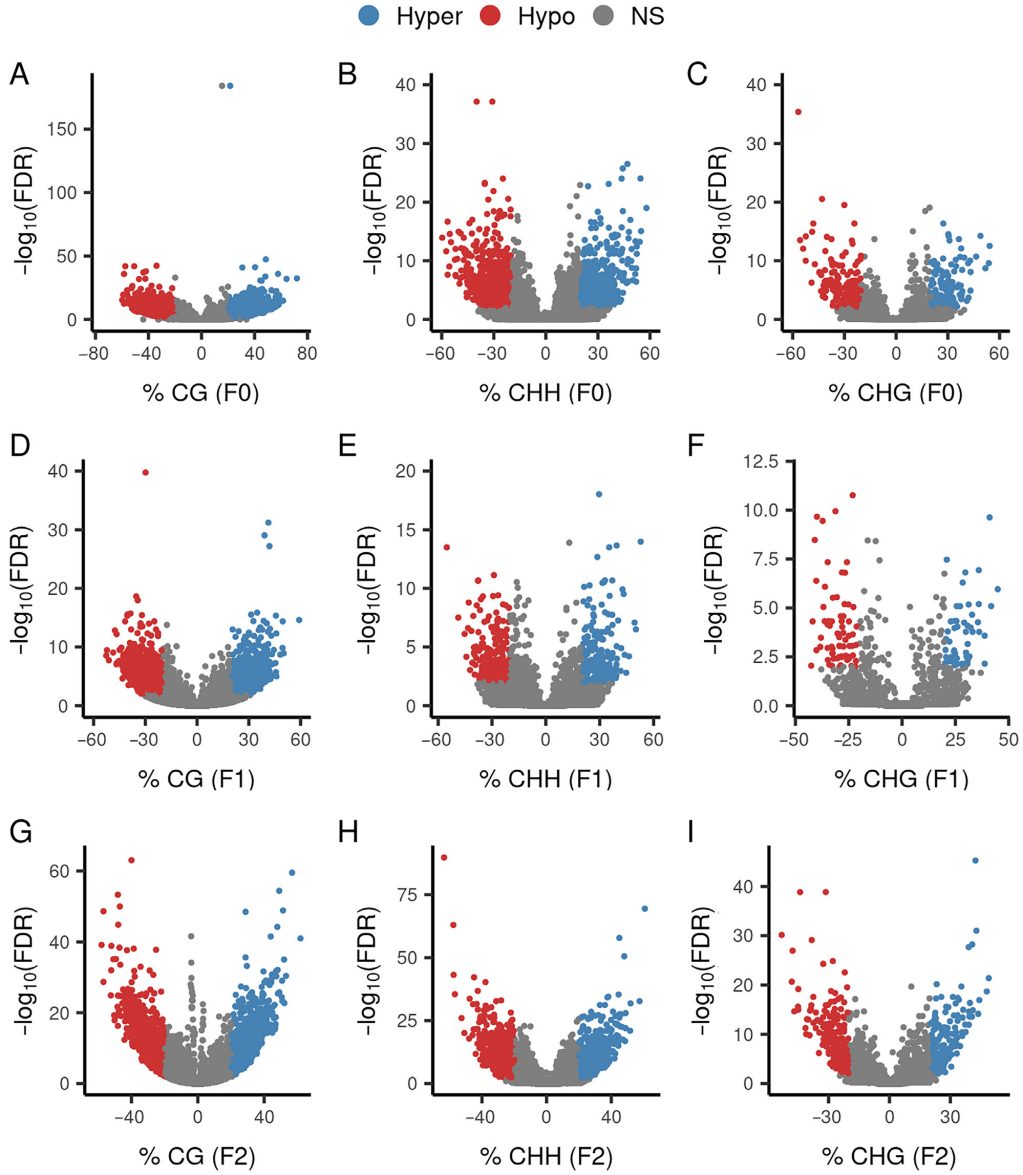
Volcano plots of DMC analyses. DMCs were defined as those with methylation difference (*x-*axis) greater than 20% between the treatment and control groups and with a FDR of 0.01 (*y-*axis) as threshold values. Red and blue dots represent DMCs hypomethylated and hypermethylated in methionine-treated group compared to control group, respectively. (A) CG context for F0. (B) CHH context for F0. (C) CHG context for F0. (D) CG context for F1. (E) CHH context for F1. (F) CHG context for F1. (G) CG context for F2. (H) CHH context for F2. (I) CHG context for F2.

To address evidence of TEI, we investigated the effects of paternal diet on phenotypes and DNA methylation changes in sperm of 3 generations of sheep. To reduce genetic variation, male twin pairs (F0 generation) were used; 1 twin was supplemented with the amino acid methionine (a methyl donor) in the diet from weaning to puberty, and the other twin was used as a control. Treated and untreated F0 males were bred to untreated females to produce the F1 generation (*n* = 225). F1 male progeny of F0 treated and untreated males were bred to untreated females to produce the F2 generation (*n* = 188). All F1 and F2 animals were fed the control diet; only the F0 generation treated males were fed diets supplemented with methionine. To examine whether methionine supplementation alters DNA methylation patterns in sperm, we performed whole-genome bisulfite sequencing (WGBS) for the 10 F0 males used to produce the F1 generation. A total of 5,669 differentially methylated cytosines (DMCs), defined as cytosines with methylation level difference ≥ 20% and false discovery rate (FDR) ≤ 0.01, in the CG context (dinucleotide where a cytosine is followed by a guanine base), were found between the treatment and the control groups (Fig. 1A; Dataset S1). We also found DMCs in non-CG contexts, including 1,288 CHHs (trinucleotides where H represents A, C, or T) and 329 CHGs (Fig. 1B and C; Dataset S1).

**Fig. 2.**
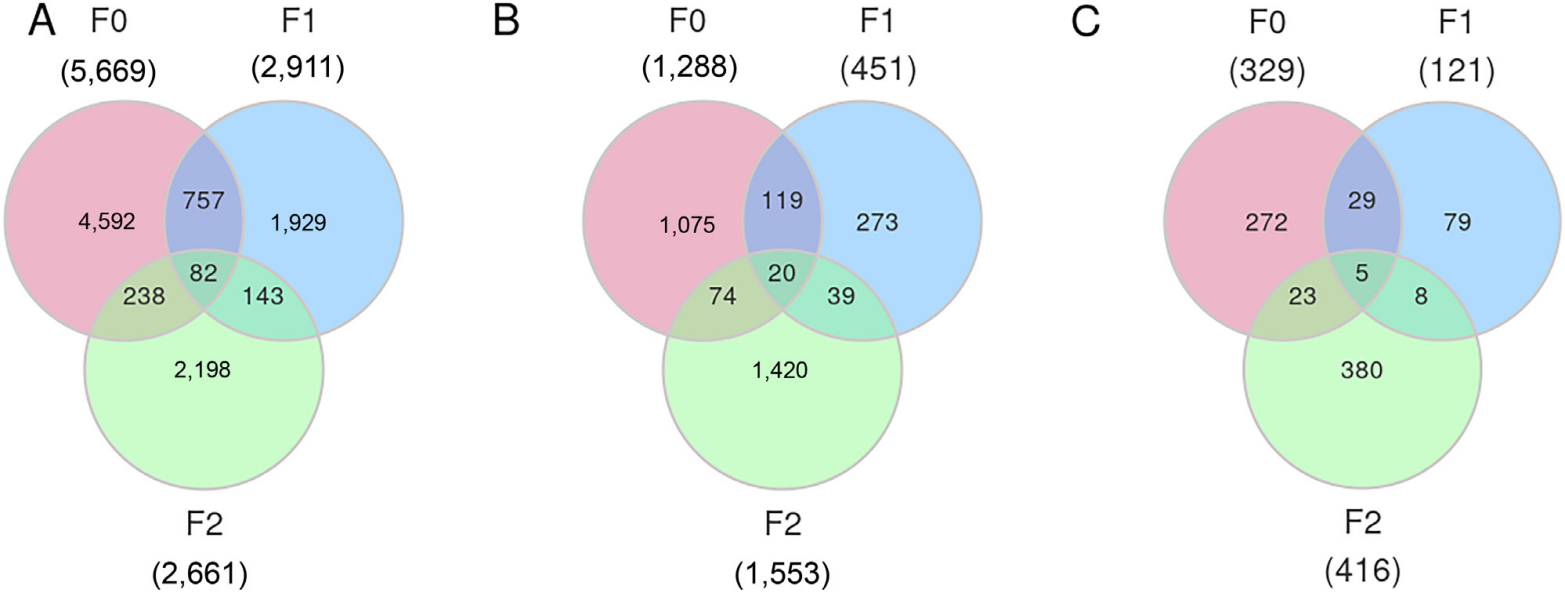
Venn diagrams of DMCs that overlapped across generations. DMCs overlap between F0, F1, and F2 generations in (A) CG, (B) CHH, and (C) CHG contexts.

To investigate whether the DMCs of the F0 generation are inherited transgenerationally, we performed WGBS of sperm from 45 F1 animals (5 pooled samples from the progeny of the F0-treated group and 5 pooled samples from the control group) and 20 individual sperm samples of males from the F2 generation (10 grand-progeny from the F0-treated group and 10 grand-progeny from the F0 control group). We detected 2,911 CG, 451 CHH, and 121 CHG DMCs in the F1 generation (Fig. 1D–F) and 2,661 CG, 1,553 CHH, and 416 CHG DMCs in the F2 generation (Fig. 1G–I). The distribution and the total number of cytosines and DMCs for CG, CHH, and CHG contexts across the genome for all 3 generations can be accessed in Supplementary Material Appendix, Figures S1–S6. There was a significant overlap of sperm DMCs between the F0 and F1 generations, including 839 CGs, 139 CHHs, and 34 CHGs (Fig. 2A–C). This intergenerational inheritance pattern was expected because the germline generating the F1 generation was also exposed to the methionine supplementation prior to puberty of the F0 generation. Similarly, a total of 225 CG, 59 CHH, and 13 CHG DMCs overlapped between the F1 and F2 generations (Fig. 2A–C). Common DMCs between only the F0 and F2 generations were also identified, indicating that some epigenetic marks appear to skip generations, as previously reported (18).

**Fig. 3.**
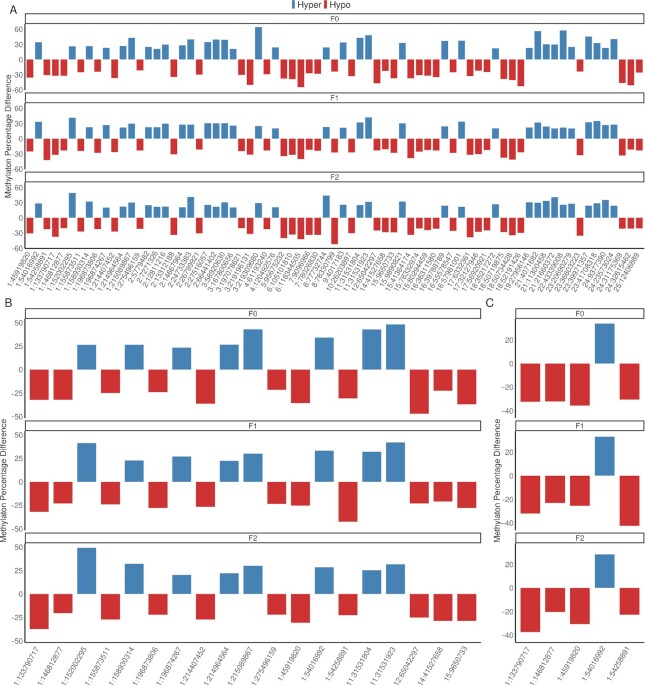
TEI marks that follow the same trend over generations. (A) A total of 72 TEI marks CG context. (B) A total of 19 TEI marks CHH context. (C) A total of 5 TEI marks CHG context. Red and blue bars represent hypomethylated and hypermethylated TEI marks (as methylation percentage difference) in the methionine-treated group compared to the control group, respectively. Top, middle, and low panels indicate F0, F1, and F2 generations, respectively. The *x*-axis represents the location of each TEI cytosine denoted as chromosome and chromosomal position in base-pair.

To meet the requirements of TEI, DNA methylation signatures altered by environmental stimuli in males must be transmitted into the F2 generation (9). Other studies have reported generation-specific DNA methylation signatures without an overlap of DMRs across generations (3, 18). Therefore, we assessed whether sperm DMCs were present across all 3 generations. Surprisingly, we identified 107 transgenerationally inherited modified cytosines; 82, 20, and 5 DMCs were in CG, CHH, and CHG contexts, respectively (Fig. 2; Dataset S2). We further investigated whether the hypermethylation and hypomethylation trends of these DMCs were maintained in the treatment groups from the F0 to the F2 generation. Interestingly, 96 of 107 transgenerationally inherited cytosines (89.7%; 72 CGs, 19 CHHs, and 5 CHGs) demonstrated the same trend of hypermethylation or hypomethylation in the treatment groups across 3 generations (Fig. 3A–C).

### Genomic locations of DMCs

To better understand the biological functions of DMCs, we determined the genomic locations of all DMC types, including CGs, CHHs, and CHGs, throughout the sheep genome (Supplementary Material Appendix, Table S1). Regardless of the cytosine context, most DMCs were located in intergenic regions (∼ 65%). About 32% of the DMCs were located in intronic regions, indicating potential involvement in regulating alternative splicing (19), while ∼ 4% of DMCs were localized to promoter regions (Supplementary Material Appendix, Table S1; Dataset S1). Only a small proportion of DMCs reside in 3’UTR (untranslated region), 5’UTR, upstream, downstream, exons, or coding regions (Supplementary Material Appendix, Table S1; Dataset S1). Studies have shown that DNA methylation in promoter and gene body regions can affect gene expression through changes in chromatin structure or transcription efficiency (20, 21). About 67% of the DMCs were mapped to repeat sequences, including the transposable elements LINEs (long interspersed nuclear elements), SINEs (short interspersed nuclear elements), and LTRs (long terminal repeats). These genomic regions constitute half of most mammalian genomes (22), and their repression relies on DNA methylation (23).

### Transgenerational inheritance of phenotypes

The concept of TEI implies that environmentally induced epigenetic changes are transmitted to unexposed subsequent generations and associated with transgenerationally inherited phenotypes. Therefore, we evaluated whether the F0 methionine-supplemented diet affected growth and reproduction phenotypes of the F1 and F2 generations. However, one argument against TEI in mammals is the challenge of ruling out the influence of genetic effects underlying the inherited phenotypes (9, 10). Thus, to avoid confounding effects, our phenotypic association analysis accounted for genetic factors including F0 twin pair and litter and nongenetic factors such as the number of siblings born, maternal age, rearing conditions, and age of the animal at the measurement. Testicular size (measured as scrotal circumference) was significantly associated with the F0 diet in both F1 (*P* = 0.032) and F2 (*P* = 0.049) generations (Table 1). The males of the methionine F1 and F2 generations that were not themselves fed the methionine diet had, on average, 1.00 and 0.81 cm smaller scrotal circumference (SC), respectively, when compared with the control groups (Table 1). Testicular size is positively correlated with male fertility and is considered an indirect measure of testicular functions and spermatogenesis (24). The growth traits of weaning weight (WWT) and postweaning weight (PWT) were significantly affected by F0 paternal diet only in F2 generation females (*P* = 0.020, *P* = 0.029, respectively). Similarly, loin muscle depth (LMD), a growth-related phenotype, was significant (*P* = 0.003) in males of the F2 generation (Table 1). Generation and sex-specific phenotypes have also been observed in other transgenerational studies (3, 18, 25), although the underlying mechanisms are not fully understood.

**Table 1.** Estimates of F0 methionine-supplemented diet effects on F1 and F2 generation phenotypes.

Phenotype	Sex	F1 generation				F2 generation			
		*N*	Estimate	SE	*P*-value	*N*	Estimate	SE	*P*-value
WWT_(kg)_	Male	74	−0.179	0.925	0.793	77	−0.511	1.206	0.671
	Female	86	−0.590	0.773	0.427	89	−1.975	0.853	0.020*
	Both	160	−0.286	0.654	0.647	166	−1.009	0.768	0.183
PWT_(kg)_	Male	−^¥^	–		–	83	1.797	1.888	0.340
	Female	75	−2.020	1.569	0.185	82	−2.561	1.190	0.029*
	Both	–	–		–	165	−0.281	1.171	0.787
SC_(cm)_	Male	69	−1.007	0.481	0.032*	72	−0.812	0.410	0.049*
LMD_(cm)_	Male	78	−0.558	0.594	0.329	73	−2.127	0.718	0.003**

WWT, weaning weight; PWT, postweaning weight; SC, scrotal circumference; LMD, loin muscle depth; *N*, number of animals analyzed; Estimate, estimate of diet effect on treatment animals compared with control animals; and SE, estimate standard errors. *P  ≤ 0.05, and ^*^*P ≤ 0.01. ^¥^PWT records were not available.

Although there is evidence of transgenerational inheritance of phenotypes such as behavioral and metabolic effects of paternal stress (26) or male fertility effects due to exposure of gestating females to toxicants (27), defined molecular mechanisms underlying these phenotypes are missing (10). Thus, there is a need to identify environmentally altered genes that are inherited across generations and associated with the observed phenotypes (5). A total of 35 out of 107 DMCs shared across all 3 generations were mapped to 33 genes (Dataset S2). Many of the transgenerationally marked genes play roles in growth (*DIRAS3, CTNNA3, CAP2, COL19A1, LRRIQ3*, and *CDH12*) and/or male reproduction (*DIRAS3, CTNNA3, LRRIQ3, DLG2, LPAR1, AXDND1, YBX3, THOC1, GK2, CATSPER3, ZBTB20*, and *STK32B*; Supplementary Material Appendix, Table S2). Interestingly, growth and male fertility phenotypes showed significant associations with the paternal diet in the F1 and F2 generations (Table 1).

Gene expression is partially regulated by epigenetic modifications, including DNA methylation in promoter and enhancer regions. Determining the relationship between DNA methylation and gene expression in the offspring following experimental paternal nutrition in F0 will help us understand the mechanisms underlying the observed phenotypic differences. We performed RNA-Seq in the same F2 generation sperm samples used for WGBS followed by Pearson's correlation (*r*) analysis between the methylation levels of TEI DMCs (with consistent methylation patterns across all 3 generations) located within genes or in promoter regions (10 kb from transcription start sites, TSS) and the normalized expression values of the corresponding genes. The analysis identified 12 genes with expression levels correlated with DMCs (*P* ≤ 0.10, *r* ≥ 0.30; Supplementary Material Appendix, Table S3), of which 5 genes play roles in male fertility (Supplementary Material Appendix, Table S2). Sperm gene expression can also affect embryo development and offspring phenotypes (28). Therefore, these correlation results provide additional support for the possible roles of these genes in transgenerationally inherited phenotypes and could be a hypothesis-generating basis for other phenotypes not measured in this study.

## Discussion

Previous studies in mammals have provided only partial evidence of TEI for various reasons, including experimental design, sample size, selected loci used to study TEI, animal model, DNA methylation method, dose and duration of environmental exposure, and the lack of a consensus about the definition of TEI. Indeed, most environmental toxicant studies in mammals show intergenerational rather than transgenerational effects (5). These factors have led to remarkable skepticism in the scientific community about the occurrence of TEI in mammals (5, 8–11). To address these valid criticisms about TEI, we used twin pairs for treatment and control subjects and produced a large number of F1 and F2 offspring for DNA methylation and phenotypic analyses. In addition, there is a need to establish a dose level that induces transgenerational response without causing significant toxicity when studying the effects of environmental insults on TEI (29). Anway et al. (27) exposed gestating females to vinclozolin levels that were greater than expected for environmental exposures. A high dose of bisphenol-A was used to study the impacts of this chemical on male fertility (30). The potential toxicological effects of these extreme dosages may confound epigenic affects. Our study showed that a moderate nutritional treatment (3 g of methionine a day for 10–12 weeks) induced significant sperm DNA methylation and phenotypic changes that were inherited by subsequent generations. Another challenge associated with transgenerational studies in mice and rats is the short exposure window to the environmental insult (29). The germline of the mouse is susceptible to epigenetic environmental changes between embryonic days E6 and E15, whereas, in our experiment, males were exposed to the methionine supplementation for about 12 weeks, from weaning to puberty.

The use of WGBS allowed us to investigate TEI at the single-nucleotide resolution level and identify a remarkable consistency of paternal diet-induced DMCs across 3 generations compared to previous studies, where low-resolution DNA methylation methods were used. To study the effects of vinclozolin on epigenetic inheritance and male fertility in rats, Anway et al. (27) used the methylation-sensitive restriction enzymes method that revealed DNA methylation changes in only 25% of the sperm DNA samples treated with vinclozolin, and only 2 genes were tested for DNA methylation. Rahman et al. (30) used the global DNA methylation assay to study the impact of bisphenol-A on male fertility in mice. Although the exposure of F0 males was associated with DNA methylation changes in subsequent generations, specific methylated cytosines were not reported in that study. Carone et al. (13) investigated the effects of paternal low-protein diet on gene expression and DNA methylation in liver samples of the F1 generation. Interestingly, the paternal diet was correlated with an elevated expression of many genes involved in lipid and cholesterol biosynthesis. However, the authors used reduced representation bisulfite sequencing (RRBS), which represents only about 1% of the genome compared to the high resolution of WGBS used in our study. WGBS is the gold standard for comprehensive and unbiased whole-genome DNA methylation profiling. To our knowledge, this is the first study to report transgenerational inheritance of differentially methylated CHH and CHG in mammals. Non-CG methylation loci are involved in gene regulation and have been linked to the expression of DNMT3L, a DNA methyltransferase essential for the silencing of retrotransposons within repeat sequences in male germ cells (31).

An essential requirement of TEI is the transmission of environmentally induced epimutations at least to the first nonexposed generation (8, 9). Our results provided evidence of transgenerational inheritance of 96 environmentally modified cytosines that show the same trend of hypermethylation or hypomethylation in the treatment groups across 3 generations. Conversely, Constantinof et al. (25) reported generation-specific DNA methylation levels of 2 genes in guinea pigs that showed decreased methylation in 1 generation and increased methylation in other generations. The authors reported differentially expressed genes in the hippocampus between animals supplemented with synthetic glucocorticoids and controls in all 3 studied generations. However, there was no overlap between these differentially expressed genes across the generations (25). The exposure of gestating female rats to glyphosate resulted in DMRs in the sperm of treated vs. control males in F1, F2, and F3 generations (3). However, no overlapping DMRs were identified between the generations (3). Similarly, Ben Maamar et al. (18) studied the effects of vinclozolin exposure of gestating rats on DNA profiles of subsequent generations. The authors reported 44 DMRs overlapped between F2 and F3 generations, 4 DMRs overlapped between F1 and F3 generations, and 2 DMRs overlapped between F1 and F2 generations (18). No DMRs were found in common across all 3 generations. Thus, the consistent hypermethylation and hypomethylation trends of a large number of DMCs across 3 generations identified in our study is a strong indication of TEI and expands our knowledge of genomic loci that escape epigenetic reprogramming.

TEI requires an incomplete erasure of the epigenetic marks during developmental reprogramming, allowing the transfer of these marks from parents to the offspring (9). Imprinted control regions and transposable elements are known to resist epigenetic reprogramming. We found a DMC in the promoter region of the imprinted gene *DIRAS3*, also harboring a LINE-1 sequence. Effects of parental diet on the expression and DNA methylation of imprinted genes, including *DIRAS3* (27), have been reported in sheep fetal tissues (32). In addition, most of the DMCs influenced by the paternal diet in this study were mapped to repeat sequences, including the transposable elements LINEs. Transcriptional silencing of transposable elements is essential for preventing their mobilization, maintaining genomic stability, and germ cell development and integrity (23, 33, 34). Supplementation of methyl donors to gestating mice resulted in increased DNA methylation at a transposable element upstream of the agouti gene leading to altering the coat color of the offspring (16). Furthermore, DNA methylation changes in transposable elements have been associated with metabolic phenotypes such as obesity (35) and initiation and progression of different cancer types (36). Thus, the diet-induced DNA methylation observed in transposable elements is intriguing because it demonstrates the vulnerability of these elements to nutritional effects, which could result in altered phenotypic outcomes across generations. We also detected TEI DMCs outside transposable elements and imprinted control regions, indicating that other genomic regions may escape DNA methylation erasure. Interestingly, 26 and 13 TEI DMCs were located in intergenic and intron regions, respectively (Dataset S2). DNA methylation in intronic regions has been associated with the regulation of alternative splicing (19). Previous studies suggested that DNA methylation in intergenic regions regulate miRNA expression (37) and contribute to genomic stability and conservation (38). In addition, Schlesinger et al. (39) reported that enhancer activation and noncoding transcriptional output are linked to DNA methylation in intergenic regions.

Despite the 2 waves of epigenetic programming in primordial germ cells and embryos immediately after fertilization, we found many sperm methylation sites that appear to escape DNA methylation erasure and are transmitted to subsequent generations. However, a formidable challenge is required to unravel molecular links between sperm DNA methylation changes and phenotypes of somatic tissues (40). We can assume a direct link between sperm DNA methylation changes and the testicular size phenotype observed in the F1 and F2 generations. A transgenerationally transmitted testis phenotype induced by an environmental factor indicates epigenetic alteration of the male germline (27). Indeed, transgenerationally marked genes detected in this study were associated with sperm motility and concentration, testis development, spermatogenesis, abnormal sperm, and male fertility (Supplementary Material Appendix, Table S2). These factors are correlated with testicular size (24). Zhou et al. (41) reported a relationship between sperm DNA methylation and germ cell development, male infertility, piRNA pathway, spermatogenesis, and germline integrity. Therefore, the DNA methylation pattern in sperm may guide the expression of genes affecting testicular size. For the growth traits observed in the F2 generation, fetal programming is a possible link between sperm DNA methylation and these phenotypes. Epigenetic modifications in growth-related genes in sperm may contribute to fetal developmental changes, which persist into adult life (42).

In summary, here we show, for the first time, that environmentally altered epigenetic marks in sheep are transmitted to subsequent generations that have not been directly exposed to the diet that initiated the epigenetic changes. Over 100 methylated cytosines, environmentally altered in the F0 generation, were inherited by the F1 and F2 generations. These results can improve our understanding of the mechanisms of non-Mendelian inheritance. Furthermore, the F0 paternal diet was associated with growth and male fertility phenotypes in subsequent generations, a finding that could be used to predict phenotypic outcomes of environmental interventions in future generations. Our findings suggest that sperm have the plasticity to reconfigure DNA methylation signatures in response to paternal diet and pass this message to subsequent generations. We believe these results advance our understanding of the relationship between nature and nurture.

## Materials and Methods

### Study design

To study the effect of paternal diet on DNA methylation in the sperm and phenotypes across generations, 2 groups of male Polypay sheep were used (43). Briefly, 10 male twin pairs (20 sheep in total) were randomly divided into 2 groups, in which 1 animal from each pair was fed a control diet, and the other twin received the control diet plus an additional, top-dressed encapsulated methionine supplement (0.22% addition, 1.5 g;  RPM Smartamine, Adisseo, Alpharetta, GA), from weaning until puberty. Between feedings, rams were group-housed and received forage (orchard grass and alfalfa hay) and water ad libitum. Then, 10 F0 rams (5 from each group) were each housed in individual pens with a group of 8–9 untreated Polypay female sheep each, for 2 breeding cycles. F0 offspring (the F1 generation) comprised 225 animals (115 males and 110 females). A maximum of 2 lambs were raised naturally per ewe; any third or fourth lambs in a litter were removed (after 48 h of colostrum consumption) and fostered to another ewe or raised artificially. All F1 animals were fed control diets for the remainder of the trial. To produce the F2 generation, 10 F1 rams (1 each from the 5 F0 sires from the control group and from the 5 F0 sires from the treatment group) were individually housed with a group of 10 untreated Polypay ewes each, for 2 breeding cycles. The F2 generation consisted of 188 animals (94 males and 94 females), which similarly to the F1 generation, were raised naturally, fostered, or raised artificially and fed the control diet until the end of the experiment.

### Semen collection and evaluation

In TEI, the epigenetic marks altered by exposure might be transmitted to subsequent generations through the germline (44). Therefore, semen samples from animals of all generations (F0, F1, and F2) were collected and analyzed. Semen was collected via electroejaculation techniques, using the Lane Pulsator IV (Lane Manufacturing Inc., Denver, CO) as specified by Gross et al. (43). In brief, ejaculate was collected into a graduated conical vial and its volume was measured. A 30-μl portion of raw semen was removed and added to a 0.1% paraformaldehyde (PFA) solution at a 1:2 dilution, which was used later to determine semen concentration with a hemocytometer. Total sperm per ejaculate was calculated by multiplying sperm concentration of the ejaculate by the total volume. The remaining semen was transported to the laboratory in a prewarmed (37°C) semen extender, which was used to evaluate the motility using computer-assisted sperm analysis (CASA) with a Hamilton Thorne semen analyzer (Hamilton-Thorne Research, Beverly, MA). To ensure rams were pubertal and producing high-quality sperm cells for both study and breeding, we used only semen samples with at least 50 × 10^6^ sperm per ejaculate and more than 10% motility (45), as such measurements have been associated with DNA and RNA integrity (46). The remaining semen from each qualifying sample was washed with phosphate-buffered saline (PBS), pelleted by centrifugation, preserved in RNAlater, and stored at −80°C.

### WGBS of F0, F1, and F2 generations

Genomic DNA from semen samples was extracted using the Quick-DNA Miniprep Plus (Zymo Research, Irvine, CA), and then bisulfite treated with the EZ DNA Methylation-Lightning kit from Zymo Research. In total, 40 samples were selected for WGBS. The 10 F0 rams used to produce the F1 generation were individually sequenced (5 from the control group and their 5 twins from the methionine treatment group). From the F1 generation, we sequenced 10 pooled samples (from 45 sheep total), of which 5 pools included offspring of the F0 control group and 5 pools consisted of offspring of the F0 treatment group. We then individually sequenced 20 rams from the F2 generation: 10 descendants of the F0 control group and 10 of the F0 treatment group (2 sheep descended from each F0 sire). WGBS was performed at the University of Illinois, Urbana-Champagne, using Illumina NovaSeq 6000 sequencing platform, with S4 flow cell, to generate 150 bp paired-end reads and reach 25x mean coverage (Illumina, San Diego, CA). Fastq files were generated and demultiplexed with the bcl2fastq Conversion Software (version 2.20, Illumina). The number of reads generated per sample varied from 189.9 to 285.7 M with an average of 233.5 M. For each sample, quality check of raw reads was performed using FastQC software, version 0.11.8 (http://www.bioinformatics.babraham.ac.uk/projects/fastqc/) and TrimGalore version 0.6.5 (https://www.bioinformatics.babraham.ac.uk/projects/trim_galore/) was used to remove adapter sequences, and low-quality reads and bases.

### Sequence alignment and DNA methylation analysis

The cleaned data were aligned to the sheep reference genome (Oar_rambouillet_v1.0) using bowtie2 of the Bismark software, version 0.22.3 (47), followed by deduplication to remove reads aligned to the same region of the genome. On average, the percentage of alignment was 77%, varying from 67.6% to 80.7%. “Bismark_methylation_extractor” function was used to extract methylation calls at a single-base resolution (47) discriminating between cytosines in CG, CHH, or CHG context, where H is A, C, or T. Calculations of read counts and methylation levels were carried out using the “methylKit” R package (48). A minimum of 10 reads per cytosine among all samples was used as a cutoff for further analysis. DMCs were defined as those with methylation difference greater than 20% between the treatment and control groups, and with a FDR of 0.01 as threshold values. DMCs that had higher or lower methylation in treatment animals when compared with the control animals were considered as hypermethylated or hypomethylated, respectively. Exon, intron, promoter, and intergenic regions that overlapped with DMCs were annotated. Promoter regions were defined as 10 kb upstream of transcription start sites (TSSs) of genes. Upstream and downstream features were considered as 200 bp sequences from genes start and end positions, respectively. Repetitive elements were downloaded from the University of California, Santa Cruz (UCSC) database (49) and overlapped with the DMCs. Figures were generated using the R package “ggplot2” (50).

### Identification of RNAs expressed in the F2 generation sperm

The same 20 semen samples from the F2 generation used for WGBS were also used for RNA sequencing. The RNAlater-preserved samples (200 ul) were centrifuged for 4 minutes at 4,000 rpm. After supernatant removal, cells were suspended with 1 ml of somatic cell lysis buffer for 4 minutes on ice (51). Samples were centrifuged for 4 minutes at 4,000 rpm and lysis supernatant was removed. Total RNA was then extracted using 1 ml of TRIzol Reagent (Life Technologies, Carlsbad, CA), according to manufacturer instructions, followed by DNase I treatment (Lucigen, Middleton, WI). RNA quality and quantity were determined by NanodropOne (Thermofisher Scientific, Wilmington, DE) and electrophoresis before RNA sequencing (RNA-Seq). RNA-Seq was performed at the University of Illinois, Urbana-Champagne, using Illumina NovaSeq 6000 sequencing platform, generating, on average, 37.6 M (31.8–47.4 M) 100 bp single-end reads (Illumina). The bcl2fastq Conversion Software (version 2.20, Illumina) was used to generate and demultiplexe the Fastq files. For each sample, quality check of raw reads was performed using FastQC software (http://www.bioinformatics.babraham.ac.uk/projects/fastqc/), and Trimmomatic (52) was used to remove adapter sequences, and low-quality reads and bases. STAR (53) was then used to align the trimmed reads to the sheep reference genome (Oar_rambouillet_v1.0) using the “--quantMode GeneCounts” option to estimate gene counts. In total, 16,098 expressed genes with at least 15 counts in more than 10 samples were considered for further analysis. The R package “edgeR” (54) was used to normalize gene counts based on the trimmed mean of M-values (TMM) method.

### Integrating DNA methylation and gene expression

To identify genes affected by transgenerational DMCs, Pearson's correlation (*r*) was calculated between the methylation levels of DMCs located within or in promoter regions (10 kb from TSS) and the normalized expression values of the corresponding genes (55). Methylation levels were defined as the ratio of the intensities of methylated and unmethylated cytosines. Significant correlation was achieved with *P*-value ≤ 0.10 and *r* ≥ 0.30 (55).

### Phenotype collection and association analysis

To evaluate whether the F0 methionine-supplemented diet affected growth and reproduction phenotypes of the F1 and F2 generations, we performed an association analysis between F0 diet and birth weight (BWT), WWT, PWT, SC, fat depth (FD), and LMD phenotypes. SC was measured at the widest point in the scrotum using a flexible measuring tape. Each ram was prepared for ultrasound measurements by shearing a dorsal area, on the left side, over the 12th and 13th ribs, which was then rubbed with vegetable oil to create a connection medium for the ultrasound (56). The Longissimus dorsi muscle was scanned using an Aloka SSD-500 portable ultrasound machine with a 7.5 MHz linear probe and images were saved to a laptop for further analyses. Using the Image J software (57), FD was obtained by measuring adipose tissue that was between the muscle and skin, and LMD was determined at the vertically deepest point of the muscle.

Phenotypes were evaluated for each generation (F1 and F2) using linear mixed models implemented in the “lme4” R package (58). For both generations, diet was included as a fixed effect, birth type (number of siblings born) and age of the dam were included as covariables, and the F0 ram pair and litter were included as random effects in the model. Rearing type (single, naturally, artificially, or foster reared) was also included as a fixed effect, and age at measurement as a covariable in the model for all traits, except for birth weight. For LMD and SC, weight of the animal at measurement was also included as a covariate. Animals in the F1 generation were raised in different pens; therefore, pen was also included as a fixed effect in the F1 phenotypes analyses.

## Supplementary Material

pgac040_Supplemental_Files

## Data Availability

All study data are included in the article, SI Appendix, and Datasets S1 and S2. WGBS and RNA sequencing data have been deposited in NCBI's Gene Expression Omnibus and are accessible through GEO Series acession number GSE198935.

## References

[1] Liu Y . 2020. Revisiting Darwin's thoughts on environmentally induced heritable changes. Sci Total Environ. 738:139540. 32475722 10.1016/j.scitotenv.2020.139540

[2] Gapp K , et al. 2014. Implication of sperm RNAs in transgenerational inheritance of the effects of early trauma in mice. Nat Neurosci. 17:667–669. 24728267 10.1038/nn.3695 PMC4333222

[3] Kubsad D , et al. 2019. Assessment of glyphosate induced epigenetic transgenerational inheritance of pathologies and sperm epimutations: generational toxicology. Sci Rep. 9:1–17. 31011160 10.1038/s41598-019-42860-0 PMC6476885

[4] Jirtle RL , Skinner MK. 2007. Environmental epigenomics and disease susceptibility. Nat Rev Genet. 8:253–262. 17363974 10.1038/nrg2045 PMC5940010

[5] Martos SN , yee Tang W, Wang Z. 2015. Elusive inheritance: transgenerational effects and epigenetic inheritance in human environmental disease. Prog Biophys Mol Biol. 118:44–54. 25792089 10.1016/j.pbiomolbio.2015.02.011 PMC4784256

[6] Horsthemke B . 2018. A critical view on transgenerational epigenetic inheritance in humans. Nat Commun. 9:1–4. 30061690 10.1038/s41467-018-05445-5 PMC6065375

[7] Morgan HD , Santos F, Green K, Dean W, Reik W. 2005. Epigenetic reprogramming in mammals. Hum Mol Genet. 14:R47–58. 15809273 10.1093/hmg/ddi114

[8] Blanco Rodríguez J , Camprubí Sánchez C. 2019. Epigenetic transgenerational inheritance. Adv Exp Med Biol. 1166:57–74. 31301046 10.1007/978-3-030-21664-1_4

[9] van Otterdijk SD , Michels KB. 2016. Transgenerational epigenetic inheritance in mammals: how good is the evidence?. FASEB J Off Publ Fed Am Soc Exp Biol. 30:2457–2465. 10.1096/fj.201500083 27037350

[10] Grossniklaus U , Kelly B, Ferguson-Smith AC, Pembrey M, Lindquist S. 2013. Transgenerational epigenetic inheritance: how important is it?. Nat Rev Genet. 14:228–235. 23416892 10.1038/nrg3435 PMC4066847

[11] Nadeau JH . 2015. The nature of evidence for and against epigenetic inheritance. Genome Biol. 16:1–2. 26162370 10.1186/s13059-015-0709-y PMC4499204

[12] Rakyan VK , et al. 2003. Transgenerational inheritance of epigenetic states at the murine Axin(Fu) allele occurs after maternal and paternal transmission. PNAS. 100:2538–2543. 12601169 10.1073/pnas.0436776100 PMC151376

[13] Carone BR , et al. 2010. Paternally induced transgenerational environmental reprogramming of metabolic gene expression in mammals. Cell. 143:1084–1096. 21183072 10.1016/j.cell.2010.12.008 PMC3039484

[14] Sadler-Riggleman I , et al. 2019. Epigenetic transgenerational inheritance of testis pathology and Sertoli cell epimutations: generational origins of male infertility. Environ Epigenet. 5:1–18. 10.1093/eep/dvz013 PMC6736068 31528361

[15] Ng SF , et al. 2010. Chronic high-fat diet in fathers programs β-cell dysfunction in female rat offspring. Nature. 467:963–966. 20962845 10.1038/nature09491

[16] Waterland RA , Jirtle RL. 2003. Transposable elements: targets for early nutritional effects on epigenetic gene regulation. Mol Cell Biol. 23:5293. 12861015 10.1128/MCB.23.15.5293-5300.2003 PMC165709

[17] Blake GET , Watson ED. 2016. Unravelling the complex mechanisms of transgenerational epigenetic inheritance. Curr Opin Chem Biol. 33:101–107. 27327212 10.1016/j.cbpa.2016.06.008

[18] Ben Maamar M , 2018. Alterations in sperm DNA methylation, non-coding RNA expression, and histone retention mediate vinclozolin-induced epigenetic transgenerational inheritance of disease. Environ Epigenet. 4:1–19. 10.1093/eep/dvy010 PMC5920293 29732173

[19] Zheng Z , Wei X, Hildebrandt A, Schmidt B. 2016. A computational method for studying the relation between alternative splicing and DNA methylation. Nucleic Acids Res. 44:e19. 26365234 10.1093/nar/gkv906 PMC4737180

[20] Lorincz MC , Dickerson DR, Schmitt M, Groudine M. 2004. Intragenic DNA methylation alters chromatin structure and elongation efficiency in mammalian cells. Nat Struct Mol Biol. 11:1068–1075. 15467727 10.1038/nsmb840

[21] Klose RJ , Bird AP. 2006. Genomic DNA methylation: the mark and its mediators. Trends Biochem Sci. 31:89–97. 16403636 10.1016/j.tibs.2005.12.008

[22] Adelson DL , Raison JM, Edgar RC. 2009. Characterization and distribution of retrotransposons and simple sequence repeats in the bovine genome. Proc Natl Acad Sci. 106:12855–12860. 19625614 10.1073/pnas.0901282106 PMC2722308

[23] Iwasaki YW , Siomi MC, Siomi H. 2015. PIWI-interacting RNA: its biogenesis and functions. Annu Rev Biochem. 84:405–433. 25747396 10.1146/annurev-biochem-060614-034258

[24] Sakamoto H , et al. 2008. Relationship between testicular size by ultrasonography and testicular function: measurement of testicular length, width, and depth in patients with infertility. Int J Urol. 15:529–533. 18430152 10.1111/j.1442-2042.2008.02071.x

[25] Constantinof A , et al. 2019. Prenatal glucocorticoid exposure results in changes in gene transcription and DNA methylation in the female juvenile guinea pig hippocampus across three generations. Sci Rep. 9:1–12. 31796763 10.1038/s41598-019-54456-9 PMC6890750

[26] van Steenwyk G , Roszkowski M, Manuella F, Franklin TB, Mansuy IM. 2018. Transgenerational inheritance of behavioral and metabolic effects of paternal exposure to traumatic stress in early postnatal life: evidence in the 4th generation. Environ Epigenet. 4:1–8. 10.1093/eep/dvy023 PMC6190267 30349741

[27] Anway MD , Cupp AS, Uzumcu N, Skinner MK. 2005. Epigenetic transgenerational actions of endocrine disruptors and male fertility. Science. 308:1466–1469. 15933200 10.1126/science.1108190 PMC11423801

[28] Teltumbade M , Bhalla A, Sharma A. 2020. Paternal inheritance of diet induced metabolic traits correlates with germline regulation of diet induced coding gene expression. Genomics. 112:567–573. 30986426 10.1016/j.ygeno.2019.04.008

[29] Legoff L , D'Cruz SC, Tevosian S, Primig M, Smagulova F. 2019. Transgenerational inheritance of environmentally induced epigenetic alterations during mammalian development. Cells. 8:1559. 31816913 10.3390/cells8121559 PMC6953051

[30] Rahman S , Pang W-K, Ryu D-Y, Park Y-J, Pang M-G. 2020. Multigenerational and transgenerational impact of paternal bisphenol A exposure on male fertility in a mouse model. How does paternal exposure to bisphenol A (BPA) affect the fertility of male offspring in mice in future generations?. Hum Reprod. 35:2020. 10.1093/humrep/deaa139 32644108

[31] Patil V , Ward RL, Hesson LB. 2014. The evidence for functional non-CpG methylation in mammalian cells. Epigenetics. 9:823–828. 24717538 10.4161/epi.28741 PMC4065179

[32] Lan X , et al. 2013. Maternal diet during pregnancy induces gene expression and DNA methylation changes in fetal tissues in sheep. Front Genet. 4:49. 23577020 10.3389/fgene.2013.00049 PMC3617393

[33] Huang H , et al. 2011. piRNA-associated germline nuage formation and spermatogenesis require MitoPLD profusogenic mitochondrial-surface lipid signaling. Dev Cell. 20:376–387. 21397848 10.1016/j.devcel.2011.01.004 PMC3061402

[34] Kostova E , et al. 2007. Association of three isoforms of the meiotic BOULE gene with spermatogenic failure in infertile men. Mol Hum Reprod. 13, 85–93. 17114206 10.1093/molehr/gal101

[35] Morgan HD , Sutherland HGE, Martin DIK, Whitelaw E. 1999. Epigenetic inheritance at the agouti locus in the mouse. Nat Genet. 23:314–318. 10545949 10.1038/15490

[36] Ponomaryova AA , et al. 2020. Aberrant methylation of LINE-1 transposable elements: a search for cancer biomarkers. Cells. 9:2017. 32887319 10.3390/cells9092017 PMC7563416

[37] Pheiffer C , Erasmus RT, Kengne AP, Matsha TE. 2016. Differential DNA methylation of microRNAs within promoters, intergenic and intragenic regions of type 2 diabetic, pre-diabetic and non-diabetic individuals. Clin Biochem. 49:433–438. 26656639 10.1016/j.clinbiochem.2015.11.021

[38] Li G , et al. 2018. DNA methylation subpatterns at distinct regulatory regions in human early embryos. Open Biol. 8:180131. 30381360 10.1098/rsob.180131 PMC6223221

[39] Schlesinger F , Smith AD, Gingeras TR, Hannon GJ, Hodges E. 2013. De novo DNA demethylation and noncoding transcription define active intergenic regulatory elements. Genome Res. 23:1601–1604. 23811145 10.1101/gr.157271.113 PMC3787258

[40] Szyf M . 2014. Lamarck revisited: epigenetic inheritance of ancestral odor fear conditioning. Nat Neurosci. 17:2–4. 24369368 10.1038/nn.3603

[41] Zhou Y . 2018. Comparative whole genome DNA methylation profiling of cattle sperm and somatic tissues reveals striking hypomethylated patterns in sperm. GigaScience. 7:giy039. 29635292 10.1093/gigascience/giy039 PMC5928411

[42] Bird A . 2007. Perceptions of epigenetics. Nature. 447:396–398. 17522671 10.1038/nature05913

[43] Gross N , Taylor T, Crenshaw T, Khatib H. 2020. The intergenerational impacts of paternal diet on DNA methylation and offspring phenotypes in sheep. Front Genet. 11:1–14. 33250925 10.3389/fgene.2020.597943 PMC7674940

[44] Maamar MB , Nilsson EE, Skinner MK. 2021. Epigenetic transgenerational inheritance, gametogenesis and germline development. Biol Reprod. 105:570–592. 33929020 10.1093/biolre/ioab085 PMC8444706

[45] Mukasa-Mugerwa E , Ezaz Z. 1992. Relationship of testicular growth and size to age, body weight and onset of puberty in Menz ram lambs. Theriogenology. 38:979–988. 16727196 10.1016/0093-691x(92)90172-n

[46] Georgiadis AP , et al. 2015. High quality RNA in semen and sperm: isolation, analysis and potential application in clinical testing. J Urol. 193:352–359. 25088949 10.1016/j.juro.2014.07.107 PMC4382362

[47] Krueger F , Andrews SR. 2011. Bismark: a flexible aligner and methylation caller for bisulfite-seq applications. Bioinformatics. 27: 1571–1572. 21493656 10.1093/bioinformatics/btr167 PMC3102221

[48] Akalin A , et al. 2012. methylKit: a comprehensive R package for the analysis of genome-wide DNA methylation profiles. Genome Biol. 13, 1–9. 10.1186/gb-2012-13-10-r87 PMC3491415 23034086

[49] Karolchik D , et al. 2004. The UCSC table browser data retrieval tool. Nucleic Acids Res. 32:D493–D496. 14681465 10.1093/nar/gkh103 PMC308837

[50] Wickham H . 2016. ggplot2: elegant graphics for data analysis. New York (NY): Springer-Verlag.

[51] Goodrich R , Johnson G, Krawetz SA. 2007. The preparation of human spermatozoal RNA for clinical analysis. Arch Androl. 53:161–167. 17612875 10.1080/01485010701216526

[52] Bolger AM , Lohse M, Usadel B. 2014. Genome analysis trimmomatic: a flexible trimmer for Illumina sequence data. Bioinformatics. 30, 2114–2120. 24695404 10.1093/bioinformatics/btu170 PMC4103590

[53] Dobin A , et al. 2013. STAR: ultrafast universal RNA-seq aligner. Bioinformatics. 29:15–21. 23104886 10.1093/bioinformatics/bts635 PMC3530905

[54] Robinson MD , Mccarthy DJ, Smyth GK. 2010. edgeR: a bioconductor package for differential expression analysis of digital gene expression data. Bioinformatics. 26:139–140. 19910308 10.1093/bioinformatics/btp616 PMC2796818

[55] Xu W , et al. 2019. Integrative analysis of DNA methylation and gene expression identified cervical cancer-specific diagnostic biomarkers. Sig Transduct Target Ther. 4:1–11. 10.1038/s41392-019-0081-6 PMC6908647 31871774

[56] Hopkins DL , Ponnampalam EN, Warner RD. 2008. Predicting the composition of lamb carcases using alternative fat and muscle depth measures. Meat Sci. 78:400–405. 22062458 10.1016/j.meatsci.2007.07.002

[57] Abràmoff MD , Magalhães PJ, Ram SJ. 2005. Image processing with ImageJ Part II. Biophoton Int. 11:36–43.

[58] Bates D , Mächler M, Bolker BM, Walker SC. 2015. Fitting linear mixed-effects models using lme4. J Stat Softw. 67. DOI: 10.18637/jss.v067.i01.

