# Supplementary material for: Paternal diet induces transgenerational epigenetic inheritance of DNA methylation signatures and phenotypes in sheep model
Source: PNAS Nexus. 2022 Apr 14;1(2):pgac040. doi: 10.1093/pnasnexus/pgac040 (PMC9802161; doi:10.1093/pnasnexus/pgac040)
Supplement: pgac040_Supplemental_Files [file pgac040_supplemental_files.zip › PNASNEXUS-PNASNEXUS-2022-00106-T-s01.docx]

**Supplementary Information for**

Paternal diet induces transgenerational epigenetic inheritance of DNA methylation signatures and phenotypes in sheep model.

Camila U. Braz, Todd Taylor, Hadjer Namous, Jessica Townsend, Thomas Crenshaw, Hasan Khatib*

*Hasan Khatib.

**Email:**  [hkhatib@wisc.edu](mailto:hkhatib@wisc.edu)

**This PDF file includes:**

Figures S1 to S6

Tables S1 to S3

Legends for Datasets S1 to S2

SI References

**Other supplementary materials for this manuscript include the following:**

Datasets S1 to S2


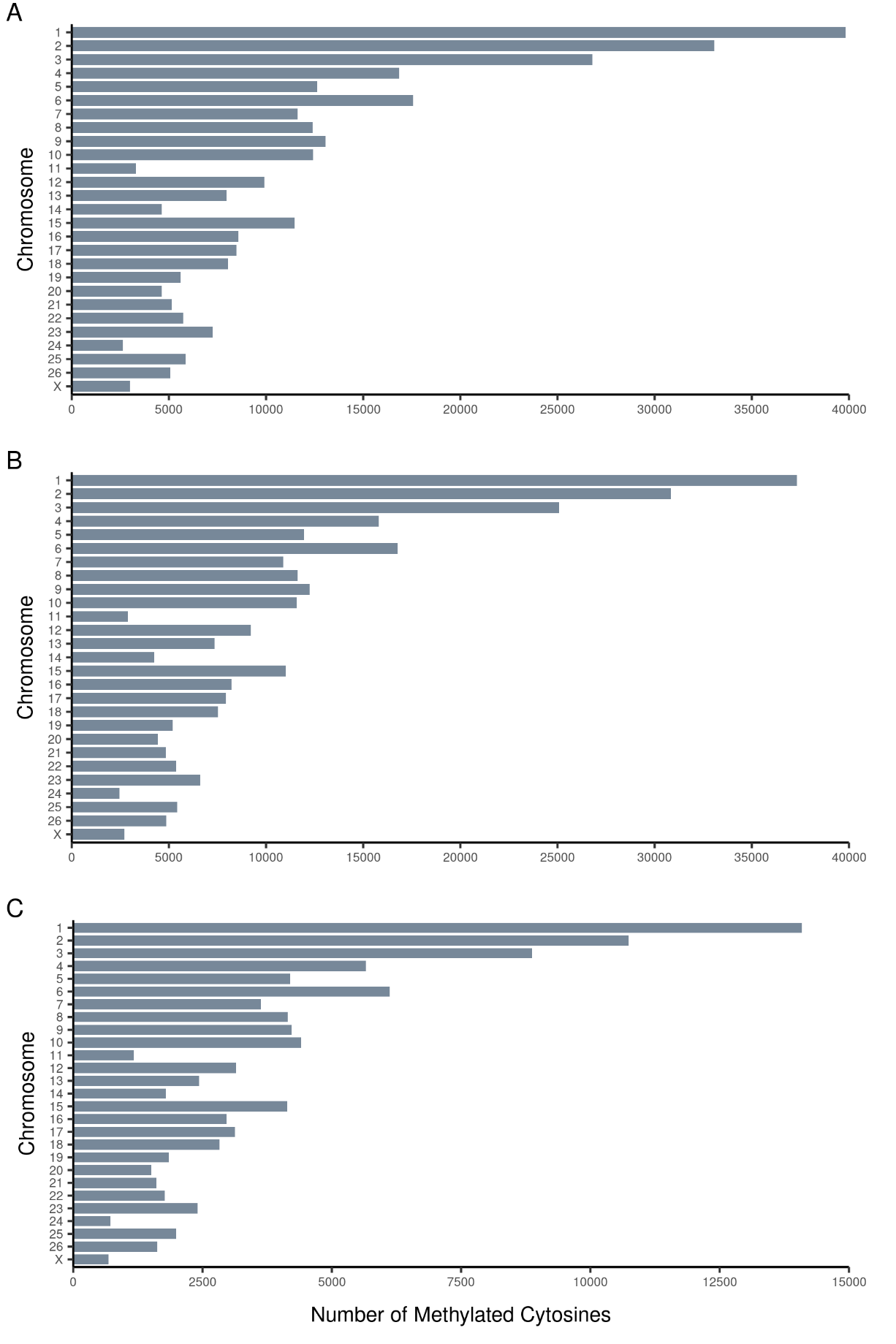


Fig. S1. Distribution of methylated cytosines in CG contexts detected in sperm across the sheep genome in the F0 (*A*), F1 (*B*), F2 (*C*) generations.


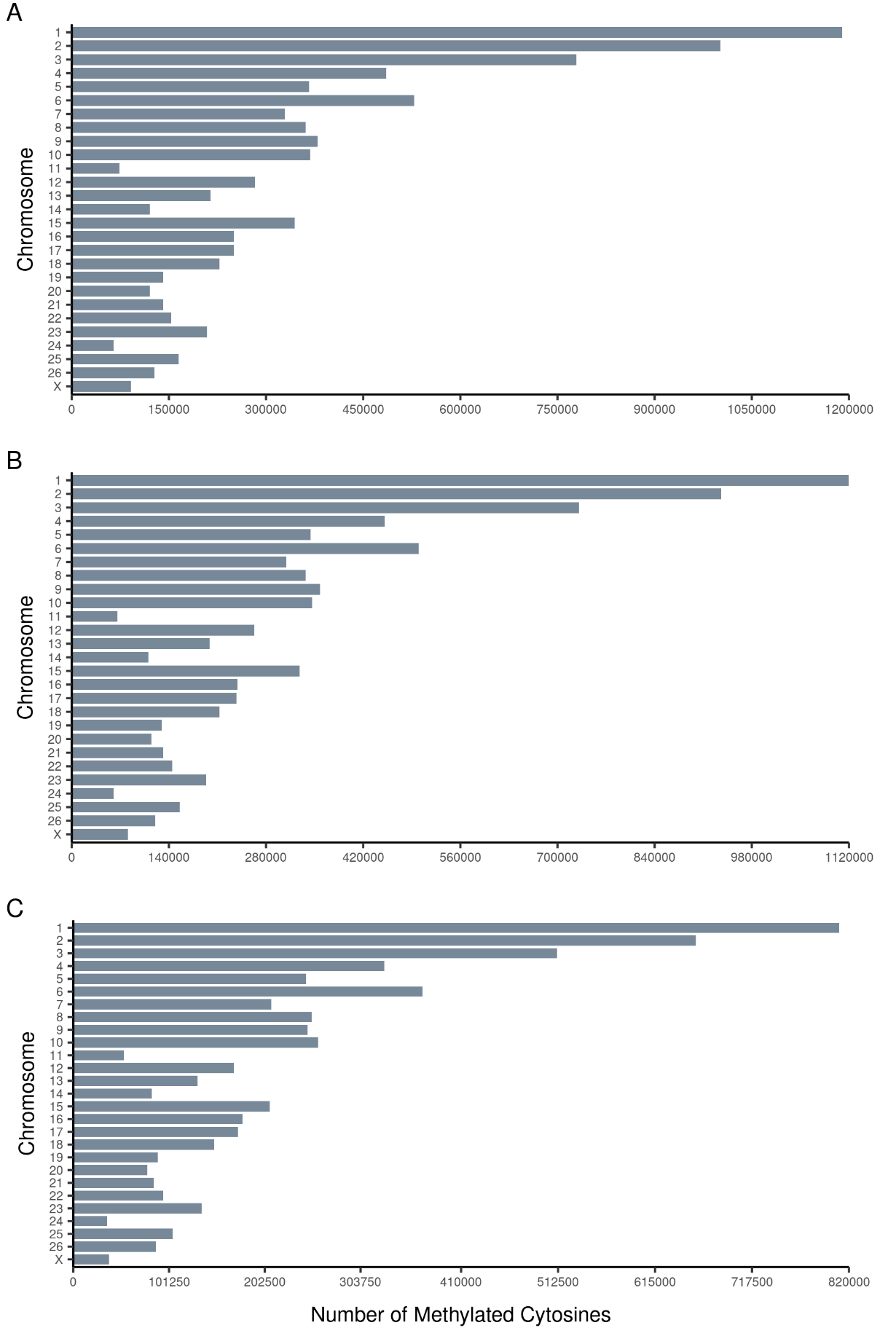


Fig. S2. Distribution of methylated cytosines in the CHH contexts detected in sperm across the sheep genome in the F0 (*A*), F1 (*B*), F2 (*C*) generations.


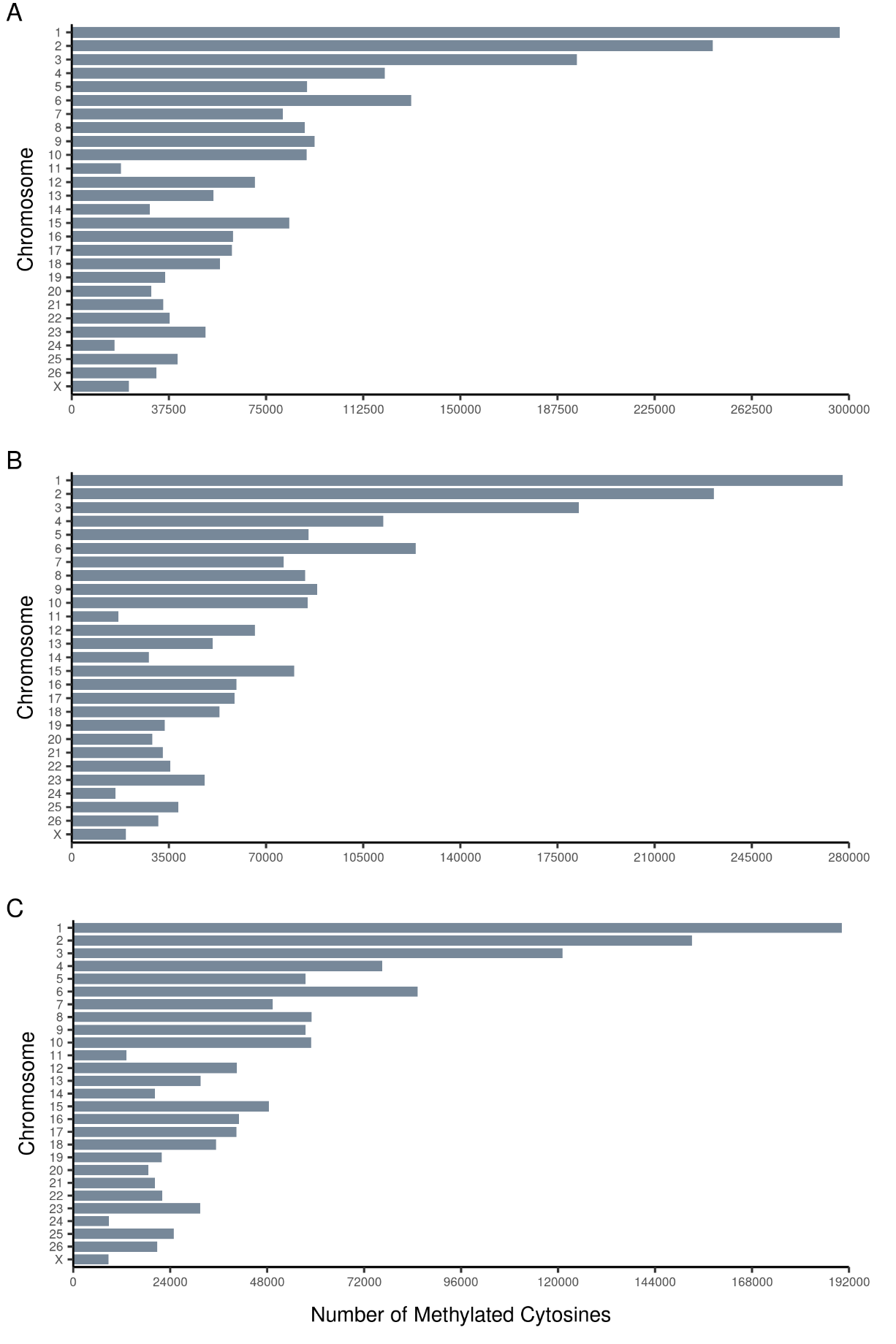


Fig. S3. Distribution of methylated cytosines in the CHG contexts detected in sperm across the sheep genome in the F0 (*A*), F1 (*B*), F2 (*C*) generations.


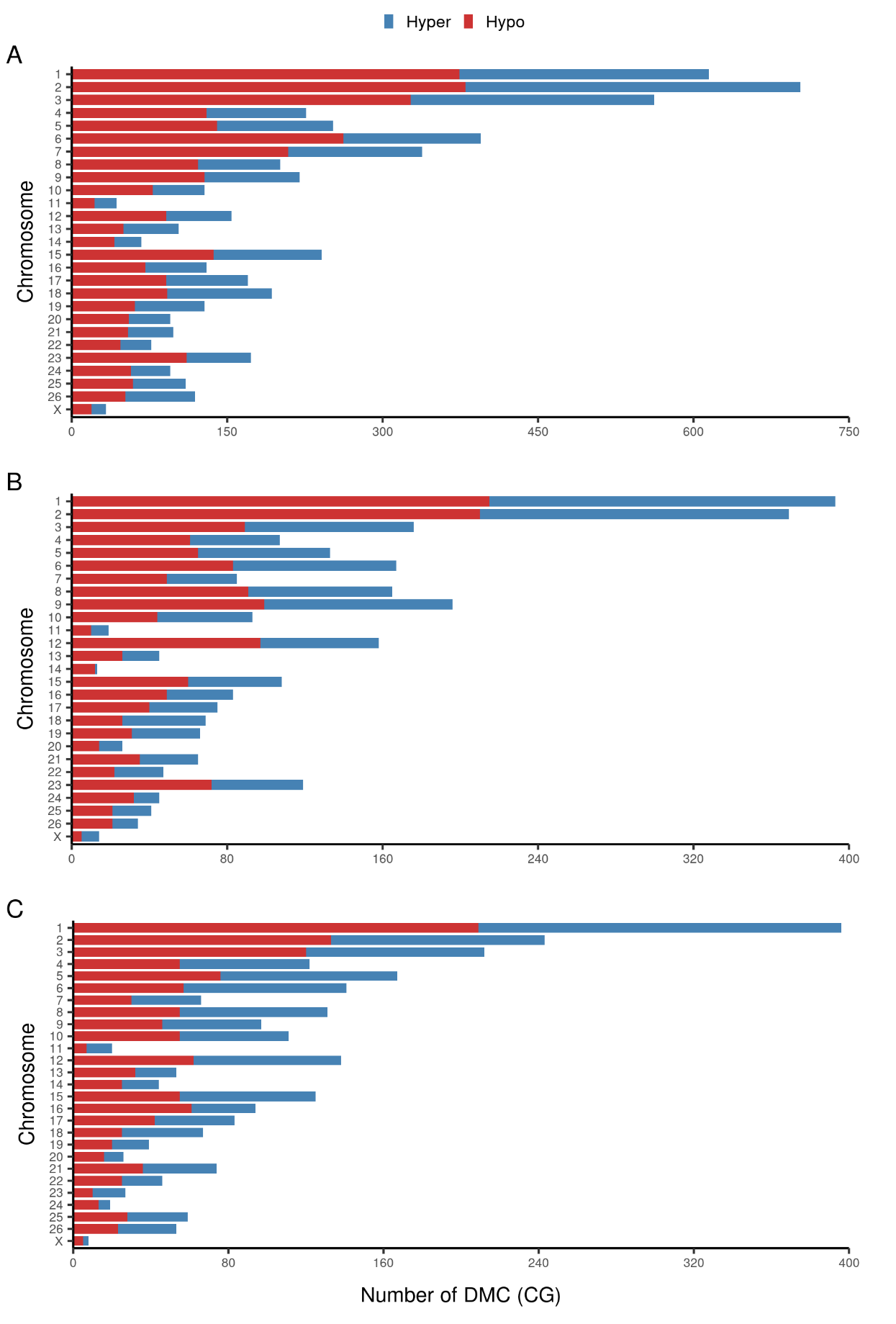


Fig. S4. Distribution of DMC (CG context) across the sheep genome in sperm of the F0 (*A*), F1 (*B*), F2 (*C*) generations, where red indicates hypomethylated and blue indicates hypermethylated in the treatment sheep compared to control.


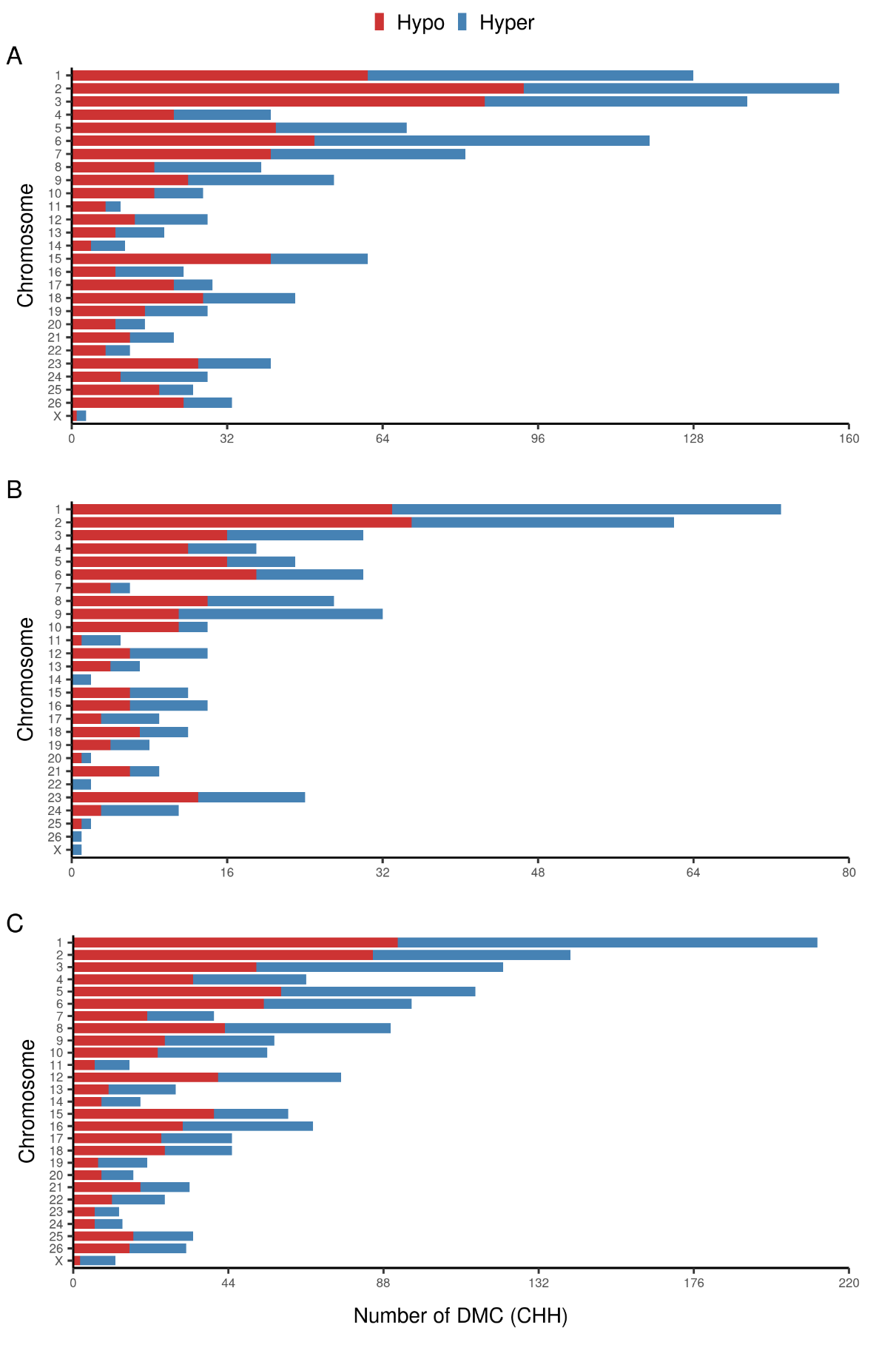


Fig. S5. Distribution of DMC (CHH context) across the sheep genome in sperm of the F0 (*A*), F1 (*B*), F2 (*C*) generations, where red indicates hypomethylated and blue indicates hypermethylated in the treatment sheep compared to control.


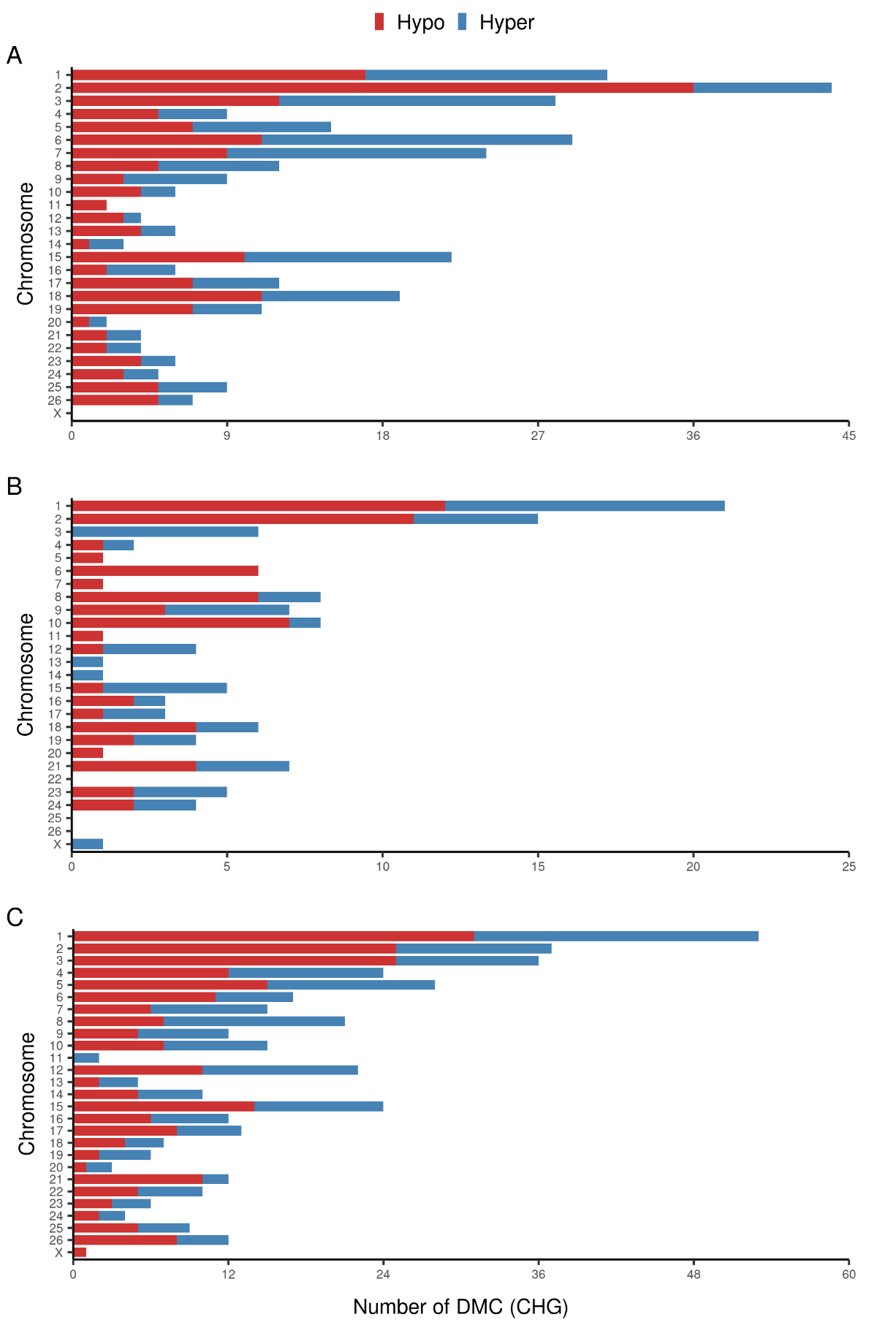


Fig. S6. Distribution of DMC (CHG context) across the sheep genome in sperm of the F0 (*A*), F1 (*B*), F2 (*C*) generations, where red indicates hypomethylated and blue indicates hypermethylated in the treatment sheep compared to control.

Table S1. Distribution numbers of differentially methylated cytosines (DMCs) for different genomic locations in CG, CHH, and CHH contexts in sperm across three generations of sheep.

| Annotation | F0 | | | F1 | | | F2 | | |
| --- | --- | --- | --- | --- | --- | --- | --- | --- | --- |
|  | CG | CHH | CHG | CG | CHH | CHG | CG | CHH | CHG |
| *Genome regions* | | | | | | | | | |
| Promoter | 235 | 40 | 14 | 120 | 13 | 7 | 131 | 72 | 26 |
| 3’UTR | 10 | 1 | 1 | 9 | - | - | 2 | 5 | 1 |
| 5’UTR | 4 | - | 1 | 2 | 1 | - | 2 | 2 | - |
| Upstream | 1 | - | - | 1 | - | 1 | 1 | - | 1 |
| Downstream | 4 | - | - | 2 | - | - | 1 | 1 | - |
| Exon | 15 | 1 | 3 | 13 | 1 | - | 7 | 7 | 3 |
| Intron | 1860 | 422 | 113 | 961 | 149 | 42 | 777 | 461 | 129 |
| CDS | 3 | - | 1 | 3 | - | - | 3 | - | 2 |
| Intergenic | 3667 | 842 | 209 | 1871 | 295 | 76 | 1806 | 1048 | 267 |
| *Transposable and Repetitive Elements* | | | | | | | | | |
| LINE | 3813 | 867 | 205 | 1959 | 306 | 82 | 1457 | 830 | 229 |
| SINE | 147 | 20 | 14 | 61 | 1 | 2 | 17 | 15 | 3 |
| LTR | 24 | 4 | 1 | 12 | - | 1 | 13 | 4 | 3 |
| Simple repeat | 30 | 12 | 5 | 18 | 8 | 2 | 33 | 16 | 6 |
| Low complexity | 24 | 3 | 4 | 10 | 4 | 2 | 23 | 12 | 5 |
| DNA | 20 | 5 | 2 | 13 | 1 | - | 18 | 13 | 5 |
| Total | 5669 | 1288 | 329 | 2911 | 451 | 121 | 2661 | 1553 | 416 |

UTR, untranslated region; CDS, coding sequence; LINE, long interspersed nuclear element; SINE, short interspersed nuclear element; LTR, long terminal repeat. Promoter, 10 kb up upstream of transcription start sites of genes; Upstream and downstream, 200 bp sequence from genes start and end positions, respectively. H is A, C, or T nucleobase.

Table S2. Biological functions of genes with transgenerationally inherited differentially methylated cytosines in sperm.

| Gene Name | Genome Location | Phenotype | Source |
| --- | --- | --- | --- |
| *Male reproduction traits* | | | |
| *DIRAS3* | Chr1:45,907,717-45,910,141 | Abnormal human sperm | (1) |
| *CTNNA3* | Chr25: 22,896,302-25,143,518 | Fertility in male swine | (2) |
| *LRRIQ3* | Chr1:53,964,907-54,160,804 | Delayed puberty in human and mouse | (3) |
| *DLG2* | Chr21:10,897,315-12,643,369 | Male puberty in mouse | (4) |
| *LPAR1* | Chr2:12,720,449-12,885,042 | Total motility and sperm membrane integrity in bovine | (5) |
| *AXDND1* | Chr12: 65,003,699-65,079,785 | Mobility of the sperm flagellum in human | (6) |
| *YBX3* | Chr3:219,162,604-219,188,095 | Defects in spermatid differentiation and male infertility in mouse | (7) |
| *THOC1* | Chr23:39,957,839-39,994,273 | Testes development in mouse | (8) |
| *GK2* | Chr6:104,142,971-104,144,635 | Spermatogenesis in mouse | (9) |
| *CATSPER3* | Chr5:48,469,580-48,504,199 | Sperm motility in mouse | (10) |
| *ZBTB20* | Chr1:196,621,742-197,129,028 | Cryptorchidism in human | (11) |
| *STK32B* | Chr6:114,061,993-114,448,338 | Sperm concentration in bovine | (12) |
| *Growth traits* | | | |
| *DIRAS3* | Chr1:45,907,717-45,910,141 | Stature in mouse | (13) |
| *CTNNA3* | Chr25: 22,896,302-25,143,518 | Body weight, body height, body length and chest circumference in sheep | (14) |
| *CAP2* | Chr20: 43,179,659-43,327,374 | Body height in sheep | (14) |
| *COL19A1* | Chr9:3,974,073-4,214,739 | Muscle development in sheep | (15) |
| *LRRIQ3* | Chr1:53,964,907-54,160,804 | Muscle development in swine | (16) |
| *CDH12* | Chr16:55,688,082-56,027,551 | Growth traits in chicken | (17) |
| *STYK1* | Chr3:219200941-219253714 | Growth in chicken | (18) |

Table S3. Description of transgenerationally inherited differentially methylated cytosines (DMC) correlated with gene expression values in sperm.

| DMC | C context | Gene | Location | TE | Correlation | P-value |
| --- | --- | --- | --- | --- | --- | --- |
| 21:11,300,458 | CG | *LOC114110160* | Intron 3 | LINE-1 | 0.572 | 0.008 |
| 4:120,492,576 | CG | *CNTNAP2* | Intron 11 | LINE-1 | -0.453 | 0.045 |
| 2:12,811,216 | CG | *LPAR1* | Intron 1 | LINE-1 | 0.451 | 0.046 |
| 1:214,964,564 | CG | *LOC105602588* | Intron 1 | LINE-1 | 0.436 | 0.055 |
| 12:65,042,297 | CG | *AXDND1* | Intron 17 | - | 0.432 | 0.057 |
| 3:219,196,131 | CG | *YBX3* | Downstream | RTE-BovB | 0.430 | 0.058 |
| 3:219,196,131 | CG | *STYK1* | Upstream | RTE-BovB | 0.414 | 0.070 |
| 1:215,089,867 | CG | *IL1RAP* | Intron 2 | LINE-1 | 0.390 | 0.089 |
| 23:39,971,357 | CG | *THOC1* | Intron 9 | - | 0.381 | 0.097 |
| 3:219,740,146 | CHH | *LOC105613000 (CD94-like)* | Promoter | - | 0.515 | 0.020 |
| 3:219,740,146 | CHH | *LOC101116641 (**CD94-like)* | Promoter | - | 0.449 | 0.047 |
| 5:48,477,588 | CHH | *PCBD2* | Downstream | LINE-1 | 0.402 | 0.079 |

Promoter, 10 kb up upstream of transcription start sites of genes; Upstream and downstream, 200 bp sequence from genes start and end positions, respectively; TE, transposable element; LINE, long interspersed nuclear element; RTE, retrotransposable element; Correlation, Pearson’s correlation between the methylation levels of DMCs and the normalized expression values of the genes. H is A, C, or T nucleobase.

Dataset S1 (separate file). Results of differentially methylated cytosine (DMC) analysis. List of all DMCs identified in sperm of F0 methionine-supplemented diet animals compared to F0 control, and their subsequent generations (F1 and F2) in CG, CHH, and CHG contexts.

Dataset S2 (separate file). Transgenerational epigenetic inherited (TEI) differentially methylated cytosines (DMC). List of the TEI DMC detected overlapping the F0, F1, and F2 generations in CG, CHH, and CHG.

**SI References**

1. S. Houshdaran, *et al.*, Widespread epigenetic abnormalities suggest a broad DNA methylation erasure defect in abnormal human sperm. *PLoS ONE* **2** (2007).

2. M. Alvarez-Rodriguez, *et al.*, The Transcriptome of Pig Spermatozoa, and Its Role in Fertility. *International Journal of Molecular Sciences* **21**, 1572 (2020).

3. S. R. Howard, *et al.*, IGSF10 mutations dysregulate gonadotropin-releasing hormone neuronal migration resulting in delayed puberty. *EMBO molecular medicine* **8**, 626–642 (2016).

4. Y. H. Jee, *et al.*, DLG2 variants in patients with pubertal disorders. *Genetics in Medicine* **22**, 1329–1337 (2020).

5. P. Brym, S. Kamiński, Microarray analysis of differential gene expression profiles in blood cells of naturally BLV-infected and uninfected Holstein–Friesian cows. *Molecular Biology Reports* **44**, 109–127 (2017).

6. Y. Vandenbrouck, *et al.*, Looking for Missing Proteins in the Proteome of Human Spermatozoa: An Update. *Journal of Proteome Research* **15**, 3998–4019 (2016).

7. E. Snyder, *et al.*, Compound Heterozygosity for Y Box Proteins Causes Sterility Due to Loss of Translational Repression. *PLoS Genetics* **11**, 1005690 (2015).

8. X. Wang, *et al.*, Thoc1 Deficiency Compromises Gene Expression Necessary for Normal Testis Development in the Mouse. *Molecular and Cellular Biology* **29**, 2794 (2009).

9. Y. Chen, *et al.*, Glycerol kinase-like proteins cooperate with Pld6 in regulating sperm mitochondrial sheath formation and male fertility. *Cell Discovery* **3**, 1–18 (2017).

10. J. Jin, *et al.*, Catsper3 and Catsper4 Are Essential for Sperm Hyperactivated Motility and Male Fertility in the Mouse. *Biology of Reproduction* **77**, 37–44 (2007).

11. S. Joseph, S. D. Mahale, Male Infertility Knowledgebase: decoding the genetic and disease landscape. *Database* **2021**, 1–14 (2021).

12. T. Suchocki, J. Szyda, Genome-wide association study for semen production traits in Holstein-Friesian bulls. *Journal of dairy science* **98**, 5774–5780 (2015).

13. Y. Yu, *et al.*, Biochemistry and Biology of ARHI (DIRAS3), an Imprinted Tumor Suppressor Gene Whose Expression Is Lost in Ovarian and Breast Cancers. *Methods in Enzymology* **407**, 455–468 (2006).

14. Z. L, *et al.*, Expression of ovine CTNNA3 and CAP2 genes and their association with growth traits. *Gene* **807** (2022).

15. R. J. Kinsella, *et al.*, Ensembl BioMarts: a hub for data retrieval across taxonomic space. *Database* **2011** (2011).

16. L. Zhang, *et al.*, Genomic variants associated with the number and diameter of muscle fibers in pigs as revealed by a genome-wide association study. *Animal* **14**, 475–481 (2020).

17. H. Zhang, *et al.*, Identification of genome-wide SNP-SNP interactions associated with important traits in chicken. *BMC genomics* **18** (2017).

18. F. Chen, *et al.*, Transcriptome Analysis of Differentially Expressed Genes Related to the Growth and Development of the Jinghai Yellow Chicken. *Genes* **10**, 539 (2019).
